# Supplementary material for: Somatic Mutations in miRNA Genes in Lung Cancer—Potential Functional Consequences of Non-Coding Sequence Variants
Source: Cancers (Basel). 2019 Jun 8;11(6):793. doi: 10.3390/cancers11060793 (PMC6627760; doi:10.3390/cancers11060793)
Supplement: Supplementary file 1 [file cancers-11-00793-s001.zip › Supplementary_Figures.docx]

**SUPPLEMENTARY FIGURES**

**Somatic mutations in miRNA genes in lung cancer – potential functional consequences of non-coding sequence variants**

Paulina Galka-Marciniak, Martyna Olga Urbanek-Trzeciak, Paulina Maria Nawrocka, Agata Dutkiewicz, Maciej Giefing, Marzena Anna Lewandowska, and Piotr Kozlowski

**
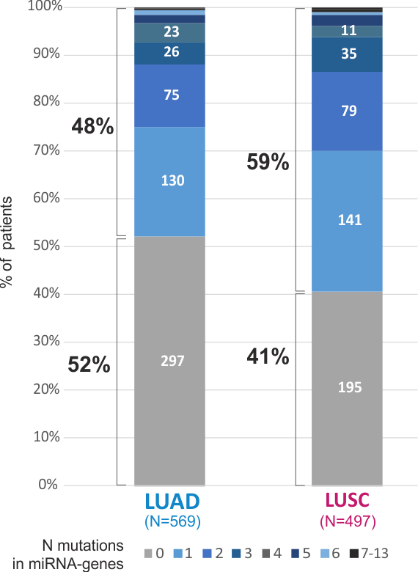
**

**Suppl. Figure S1.** Proportion of patients with mutated miRNA genes. Different colors represent the number of samples with the indicated number of mutations in LUAD and LUSC.


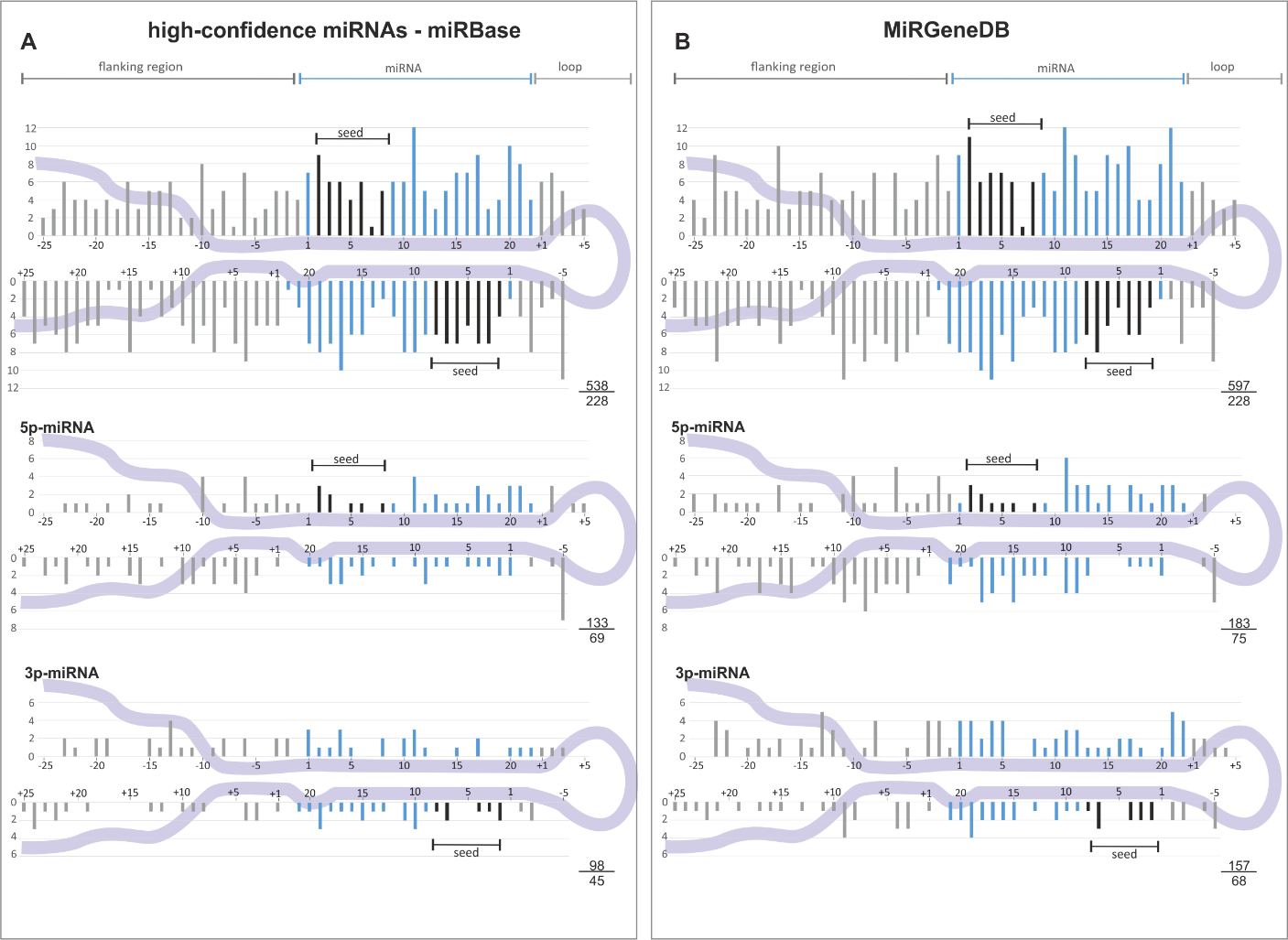


**Suppl. Figure S2**. Distribution of somatic sequence variants within precursors of high-confidence miRNAs. **A)** and **B)** High-confidence miRNAs defined in miRBase and MirGeneDB, respectively. Due to the lower number of high-confidence miRNA genes (lower total number of mutations assigned), the graphs are presented jointly for LUAD and LUSC. The scheme of the graph is shown in Figure 2.


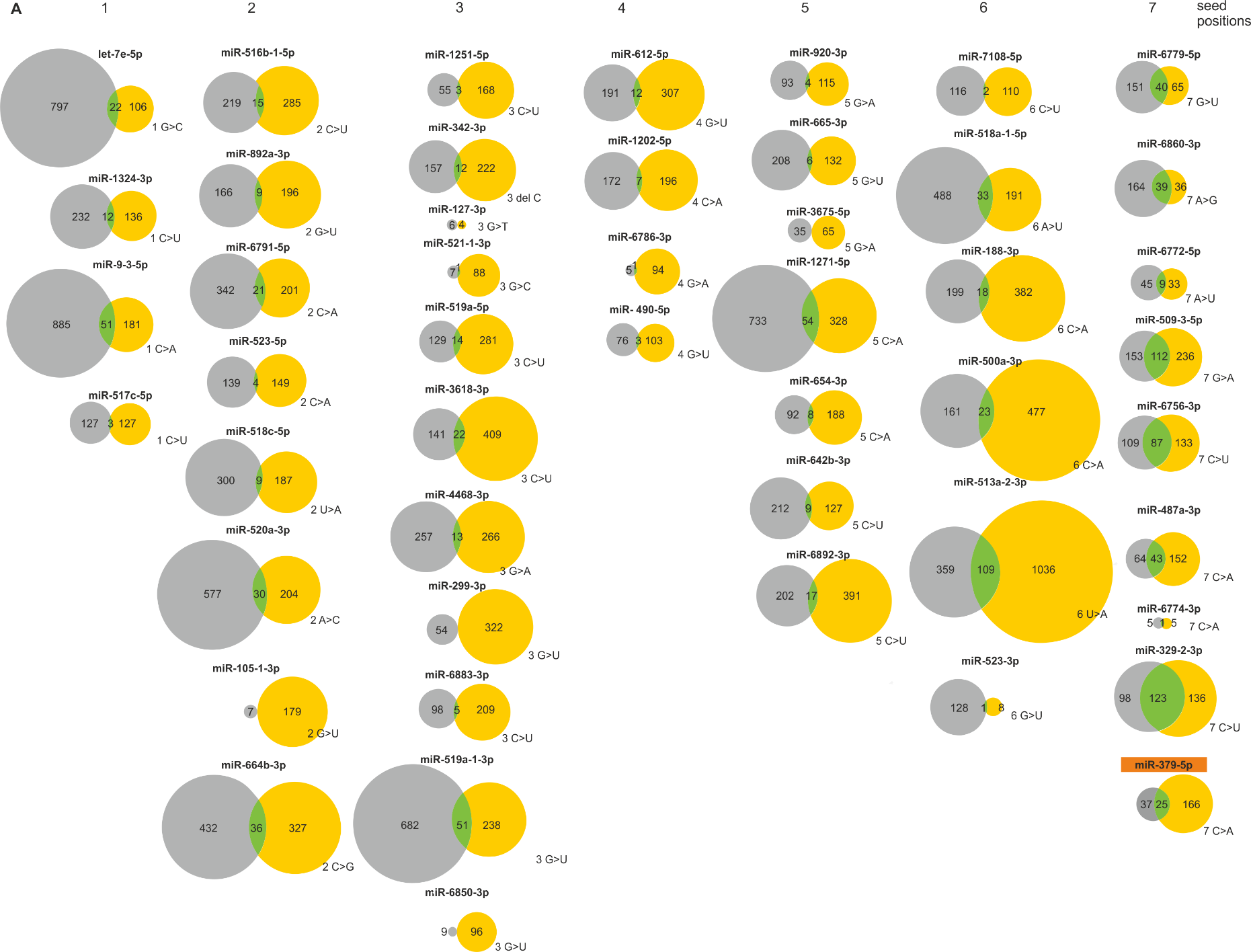


Continued on next page


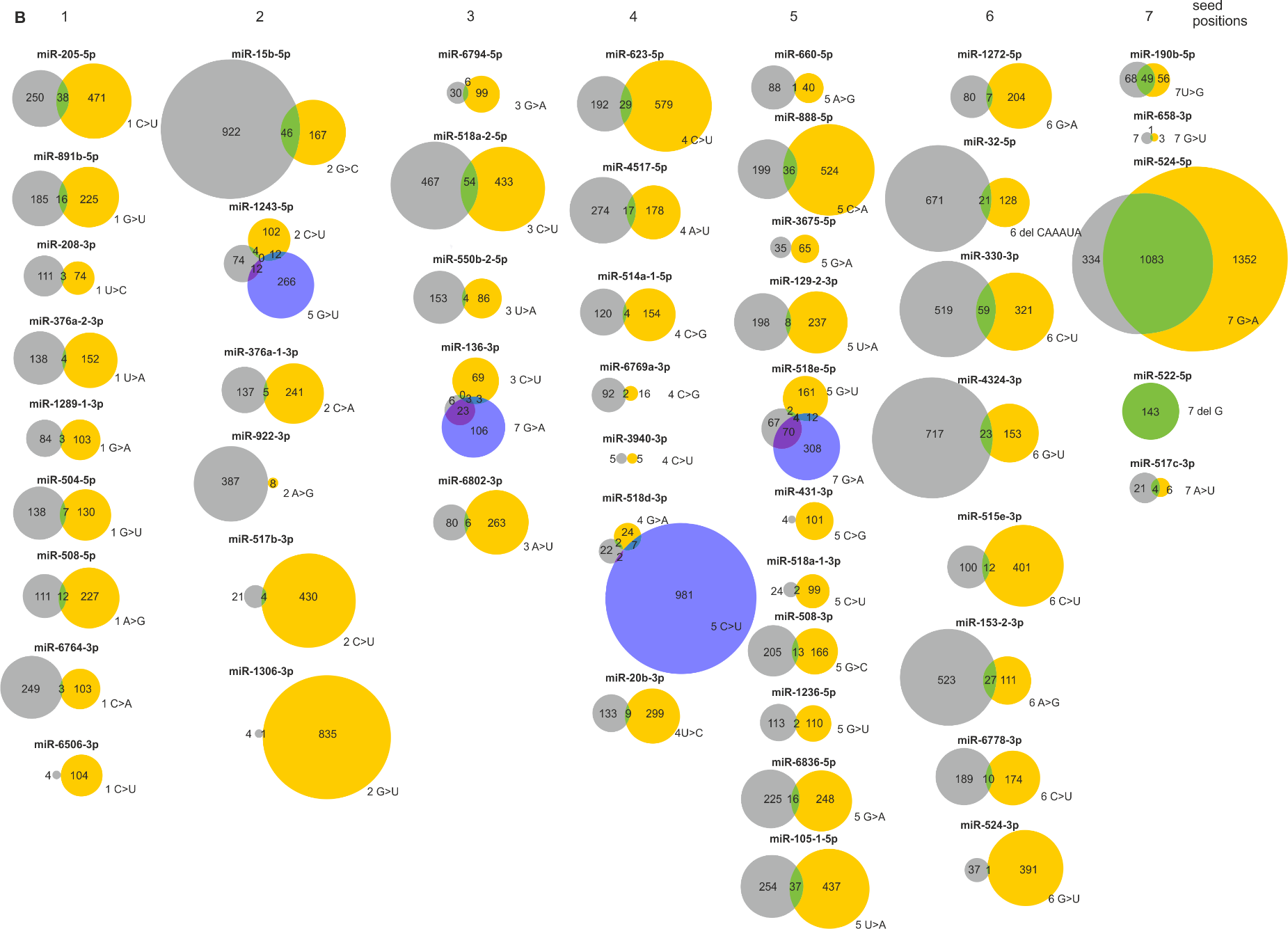


**Suppl. Figure S3**. Consequences of the seed mutations detected in **A)** LUAD and **B)** LUSC on the pool of predicted target genes. In each panel, the graphs are grouped in columns according to mutation position in the seed. The ID of miR-379-5p highlighted in orange indicates a mutation detected in the panel of lung cancer samples tested experimentally in this study. The scheme of the Venn diagrams is shown in Figure 3.

**
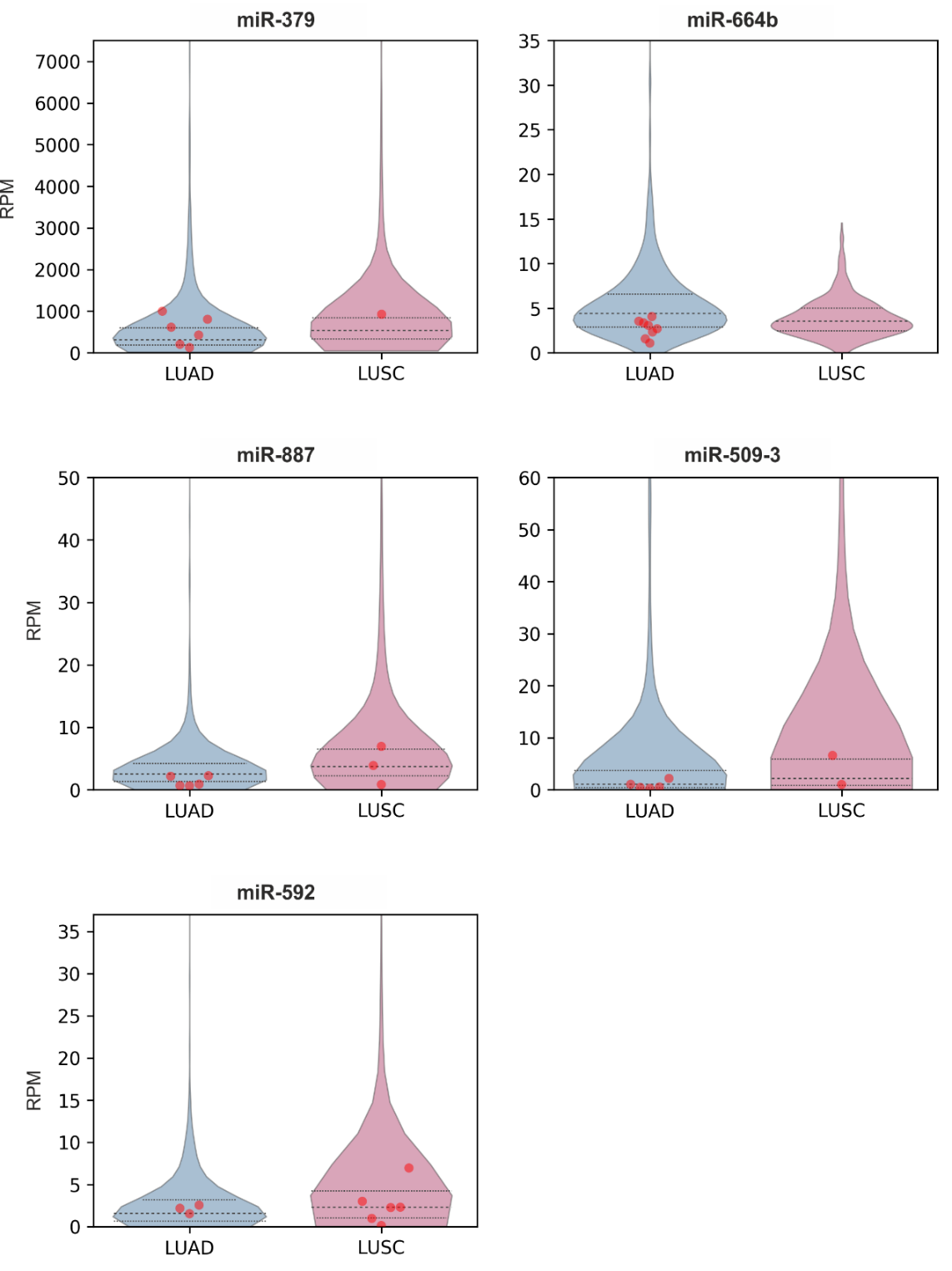
**

**Suppl. Figure S4.** Expression level of mutated hotspot miRNA genes. Violin plots show the distribution of miRNA expression levels as reads per million (RPM) in LUAD (blue) and LUSC (pink) samples. Dashed and dotted lines represent the median and 25^th^ and 75^th^ percentiles of the expression values. Red dots represent samples with a mutation in the particular hotspot miRNA gene. miRNA genes with undetermined expression or low or undetectable expression in most samples are not shown.

**
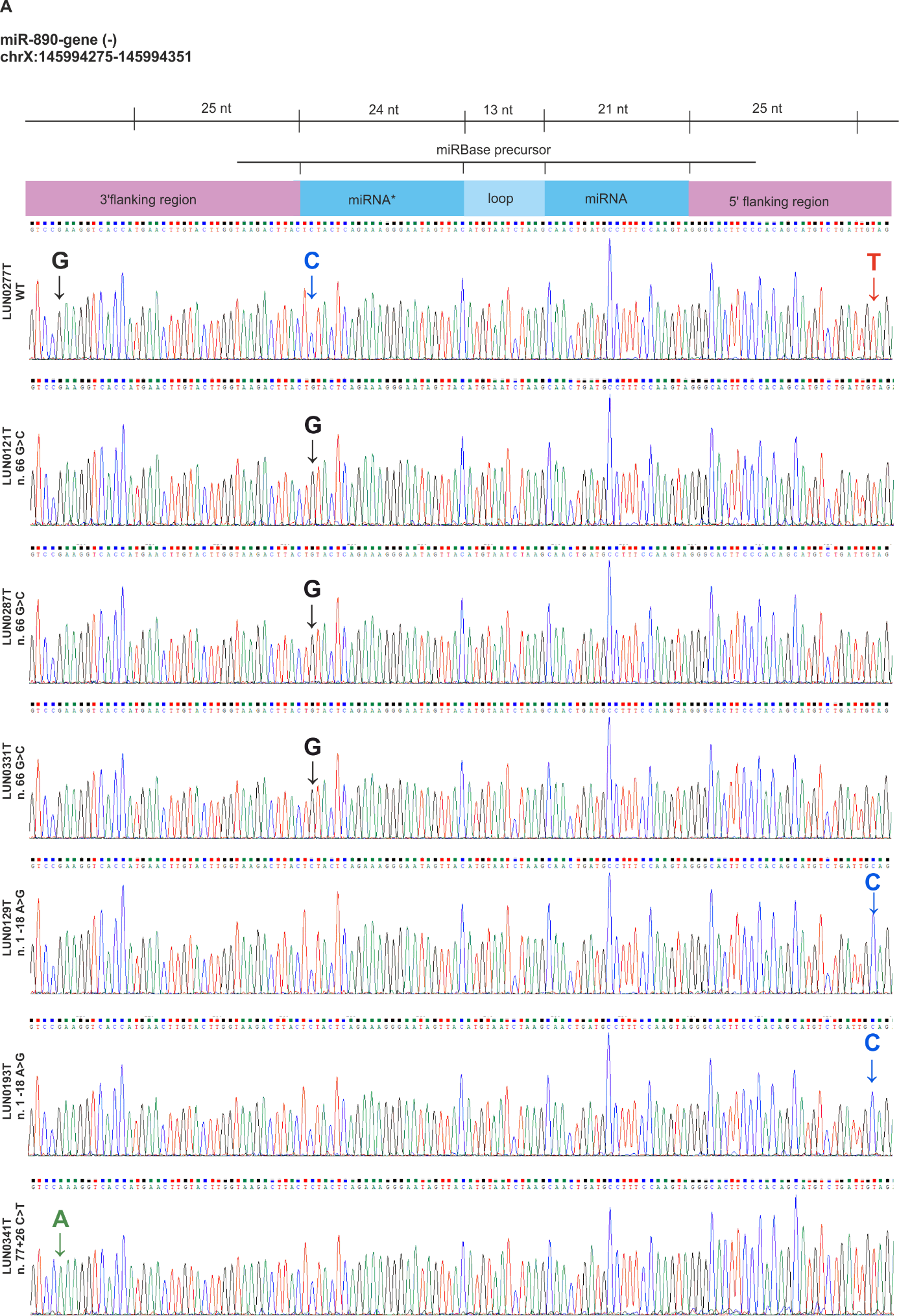
**

Continued on next page

**
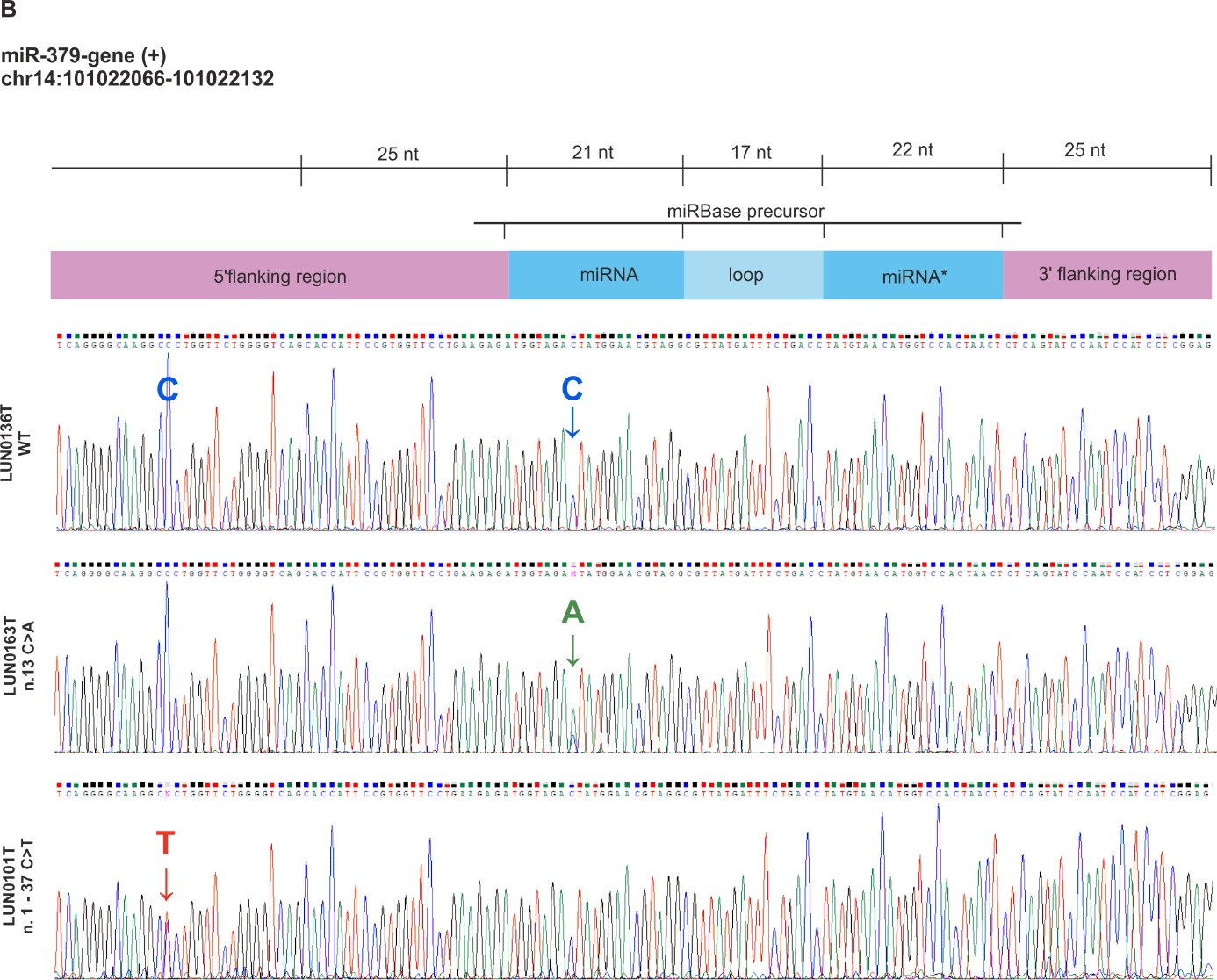
**

**Suppl. Figure S5.** miRNA gene mutations detected experimentally in a set of lung cancer samples.

**A)** and **B)** Sanger sequencing electropherograms showing sequence variants in the *miR-890* and *miR-379* genes, respectively. The upper-most electropherograms represent reference samples with wild-type sequences. The bar above the electropherograms illustrates the positions of miRNA gene subregions. Mutations in particular samples are indicated by arrowheads colored corresponding to the color of the particular nucleotide on the electropherogram. Note that the *miR-890* gene is coded by the minus strand, which results in the reverse order of the miRNA gene subregions.
